# Ambulatory blood pressure is better associated with target organ damage than clinic blood pressure in patients with primary glomerular disease

**DOI:** 10.1186/s12882-020-02200-1

**Published:** 2020-12-11

**Authors:** Ruo-wei Wen, Xiao-qiu Chen, Ye Zhu, Jian-ting Ke, Yi Du, Cheng Wang, Tan-qi Lou

**Affiliations:** 1 Division of Nephrology, Department of Medicine, the Fifth affiliated hospital Sun Yat-Sen University, ZhuHai, 519000 Guangdong China; 2 Guangdong Provincial Key Laboratory of Biomedical Imaging, the Fifth affiliated hospital Sun Yat-Sen University, Zhuhai, 519000 Guangdong China; 3 Division of Nephrology, Department of Medicine, the Third affiliated hospital Sun Yat-Sen University, GuangZhou, 510000 Guangdong China

**Keywords:** Ambulatory blood pressure, Clinic blood pressure, Target organ damage, Primary glomerular disease

## Abstract

**Background:**

Blood pressure is an important and modifiable cardiovascular risk factor. Ambulatory blood pressure monitoring (ABPM) provides valuable prognostic information in patients with chronic kidney disease (CKD), yet little is known about the association of various types of BP measurements with target organ damage (TOD) in patients with primary glomerular disease. The goal of this study was to investigate whether ambulatory blood pressure is better associated with TOD than clinic blood pressure in patients with primary glomerular disease.

**Methods:**

1178 patients with primary glomerular disease were recruited in this cross-sectional study. TOD were assessed by the following 4 parameters: left ventricular mass index (LVMI or LVH, left ventricular hypertrophy), estimated glomerular filtration rate (eGFR< 60 ml/min/1.73m^2^), albumin-to-creatinine ratio (ACR ≥ 30 mg/g) and carotid intima-media thickness (cIMT) or plaque. Receiver operating characteristic (ROC) curve and multivariate logistic regression analyses were used to evaluate the relationship between ambulatory or clinic systolic blood pressure (SBP) indexes and TOD.

**Results:**

Among 1178 patients (mean age, 39 years,54% men), 116, 458, 1031 and 251 patients had LVH, eGFR < 60 ml/min/1.73m^2^, ACR ≥ 30 mg/g and cIMT≥0.9 mm or plaque respectively. Area under ROC curves for TOD in ambulatory SBP, especially nighttime SBP, was greater than that in clinic SBP (***P*** < 0.05). Multivariate logistic regression analyses showed that 24 h SBP, daytime SBP and nighttime SBP were significantly associated with LVH, eGFR< 60 ml/min/1.73m^2^ and ACR ≥ 30 mg/g after adjustment for clinic SBP, while the association of clinic SBP was attenuated after further adjustment for nighttime SBP.

**Conclusions:**

Ambulatory blood pressure, especially nighttime blood pressure, is probably superior to clinic blood pressure and has a significant association with TOD in primary glomerular disease patients.

**Supplementary Information:**

The online version contains supplementary material available at 10.1186/s12882-020-02200-1.

## Background

Chronic kidney disease (CKD) is a worldwide public health problem [1]. In these patients, hypertension is prevalent and considered the leading risk factor for death, which contributes to 45% of male deaths and 46% of female deaths [2–4]. Hypertension is also among the most important modifiable risk factors for end-stage renal disease (ESRD). Therefore, appropriate evaluation and management of hypertension to achieve blood pressure (BP) goals in CKD patients is necessary and valuable.

Ambulatory blood pressure monitoring (ABPM) could provide detailed information on BP over a 24 h period, and it is unanimously recommended by guidelines for BP management [5–7]. Previously, we have reported the high prevalence and prognostic value of nighttime hypertension in CKD patients compared with clinic blood pressure [8–10]. Recent evidence from large-scale cohort study also suggests that higher 24-h and nighttime blood pressure measurements were significantly associated with greater risks of death and cardiovascular disease, even after adjusting for other office-based or ambulatory blood pressure measurements [11]. All these data suggested ABPM was better than clinic blood pressure when assessing target organ damage (TOD) and prognosis in CKD patients.

However, CKD patients with different etiologies, like primary glomerular disease and diabetic kidney disease, were enrolled in prior studies at the same time. Primary glomerular disease and diabetic kidney disease were two main causes of CKD in many countries. In previous studies, the percentage of patients with diabetic kidney disease or diabetes mellitus at enrollment was 11–65% [12–15]. Compared with non-diabetic kidney disease, patients with diabetic kidney disease showed different BP characteristics and had a worse prognosis [16, 17]. The systolic blood pressure control was worse and non-dipping rhythm was quite common [16]. Once in the period of massive albuminuria, the progression rate of diabetic kidney disease to ESRD is about 14 times that of other renal diseases [17], indicating patients with diabetic kidney disease would have more severe subclinical TOD, so it might be a big difference on the priority of ABPM between patients with and without diabetic kidney disease. It is very important to evaluate various types of BP measurements, especially ABPM, and assess the strength of their associations with TOD, focusing on patients with primary glomerular disease considering primary glomerular disease continues to be the very common in our country [18]. Accordingly, the objective of this study is to investigate whether ambulatory blood pressure is better associated with TOD than clinic blood pressure in patients with primary glomerular disease.

## Methods

### Study population

**Fig. 1:**
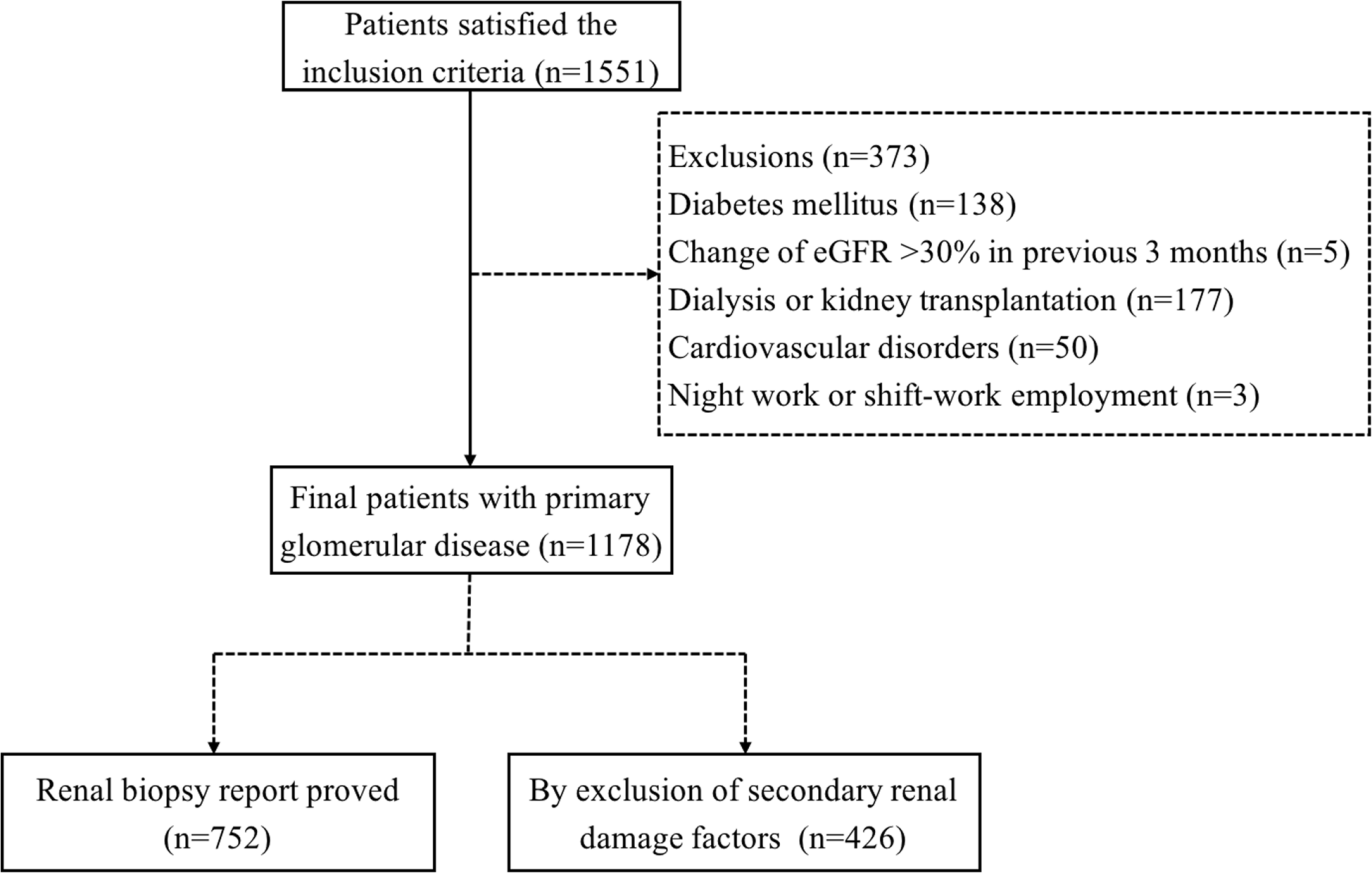
shows the flowchart of included patients

The study protocol was approved by the ethics committee of our hospital and adhered to the Declaration of Helsinki. Informed consent was obtained from each participant. Patients (14–75 years) with primary glomerular disease proved by renal biopsy or clinic findings after exclusion of secondary renal damage factors, were included. Patients were excluded from the study in case of: 1) diabetes mellitus; 2) acute changes in the eGFR > 30% in the previous 3 months; 3) maintenance dialysis or history of kidney transplantation; 4) cardiovascular disorders (unstable angina pectoris, heart failure, life-threatening arrhythmia, atrial fibrillation, stroke and grade III–IV retinopathy); 5) pregnancy; 6) night work or shift-work employment; 6) intolerance to ABPM or invalid ABPM data; 7) inability to communicate and comply with all of the study requirements; Finally, a total of 1178 patients from our hospitals were enrolled in this study (Fig. 1).

### Ambulatory and clinic blood pressure monitoring

Ambulatory blood pressure monitoring was performed with the automated measurements programmed at 15-min intervals during the daytime and 30-min intervals at night as previously [8, 19]. Appropriate cuff size was chosen based on the arm circumference and directly placed on the non-dominant arm. The monitor was programmed to measure every 15 min during the day (7:00 am to 10:00 pm), and every 30 min during the night (10:00 pm to 7:00 am). Monitoring was performed on a working day. Patients were instructed to maintain their usual but not strenuous level of activity, and to keep motionless at the time of measurement. ABPM data were invalid in cases of: 1) > 30% of measurements were lacking; 2) > 3 h data were missing, 3) sleep time at night was < 6 or > 12 h during monitoring [20].

Clinic BP was measured at the physician’s office with a standard mercury sphygmomanometer after a 5-min rest in a sitting position. For all patients, sphygmomanometric measurements were recorded by the same physician, who was not aware of the results of ABP recordings. Reported values of clinic BP were the mean of 2 or 3 measurements at 1–2 min intervals, recorded during the 2 days in which the ABPM device was installed and removed.

### Cardiac, renal and carotid assessment

Cardiac structure and function were assessed by 2 investigators trained for this purpose before starting the study. Linear measurements of interventricular septal wall thickness (IVSd), end-diastolic left ventricular internal dimension (LVIDd), and posterior wall thickness (PWTd) were obtained from M-mode tracings, using 2-dimensional echocardiography. LVM was calculated using the Duvereux method [21]. The left ventricular mass index (LVMI) was obtained by calculating the ratio of LVM to body surface area.

Concentrations of serum creatinine (Scr) were measured by an enzymatic method traceable to isotope dilution mass spectrometry. The estimated Glomerular Filtration Rate (eGFR) was calculated using 2009 Chronic Kidney Disease Epidemiology Collaboration (CKD-EPI) creatinine equation [22]. Awaking (7:00 am to 10:00 pm), bedtime (10:00 pm to 7:00 am) and 24-h urine samples were collected to predict excretion levels of urinary albumin, protein, and creatinine. Patients were asked to void their bladders at 7:00 am and 10:00 pm to ensure valid results.

Carotid intima-media thickness (cIMT) was determined by averaging 3 measurements taken on each carotid artery (in anterior, lateral and posterior directions), measuring the distance between the leading edge of the lumen–intima interface, and the leading edge of the collagenous upper layer of the adventitia using high-resolution B mode ultrasonography. Measurements were taken in areas free of obvious atherosclerotic plaques around the level of the carotid bifurcation.

### Collection of other data

Information including age, sex, height, weight, smoking and alcohol consumption status, antihypertensive medication were obtained at the time of the BP measurement. Laboratory data (hemoglobin, albumin, calcium, phosphorus, intact parathyroid hormone, triglycerides, total cholesterol, high-density lipoprotein cholesterol, low-density lipoprotein cholesterol, uric acid and blood urea nitrogen) were obtained at the initial study visit. Blood samples were taken in the morning and analyzed using a 7180 Biochemistry Autoanalyzer (Hitachi, Tokyo, Japan) with reagents from Roche Diagnostics (Mannheim, Germany).

### Definitions

CKD was divided into 5 stages and defined as the presence of kidney damage or decreased renal function (eGFR < 60 mL/min per 1.73 m2) for ≥3 months according to the Kidney Disease: Improving Global Outcomes (KDIGO) 2012 clinical practice guideline. Clinic hypertension was defined as clinic blood pressure (BP) ≥140/ 90 mmHg and ambulatory blood pressure (ABP) was defined as 24-h BP ≥130/80 mmHg. Masked hypertension was defined as a normal clinic BP (≤140/90 mmHg) and an elevated ABP (> 130/80 mmHg). White coat hypertension was regarded as increased clinic BP (> 140/90 mmHg) and normal ABP (≤130/80 mmHg). Normotension was defined as both clinic BP < 140/ 90 mmHg and ABP < 130/80 mmHg; Sustained hypertension was regarded as clinic BP ≥140/90 mmHg and ABP ≥130/80 mmHg. Nighttime hypertension was defined as nighttime systolic BP (SBP) ≥120 mmHg or/and diastolic BP (DBP) ≥70 mmHg. Isolated nighttime hypertension was defined as daytime BP < 135/85 mmHg and nighttime BP ≥120/70 mmHg. Participants with a reduction in SBP of ≥10% at night-time compared with daytime were considered to have a “dipper” pattern, and an “extreme dipper pattern” referred to a > 20% reduction at nighttime. A “non-dipper” pattern referred to a < 10% reduction at nighttime and a “reversed dipper pattern” referred to higher SBP at nighttime compared with daytime. Target organ damage (TOD) was defined if it met any of four conditions [23]: 1) left ventricular hypertrophy (LVH), namely LVMI ≥125 g/m2 (man) or ≥ 120 g/ m2 (woman); 2) eGFR < 60 mL/ min per 1.73 m^2^; 3) Urinary albumin-to-creatinine ratio (ACR) ≥30 mg/g; 4) cIMT ≥0.9 mm or existence of carotid plaque in ultrasonography.

### Statistical analysis

Statistical analysis was performed with SPSS 25.0 (IBM Corp., Armonk, NY) and Medcalc 18.9(Broekstraat, Mariakerke, Belgium). Descriptive statistics were mean ± SD for continuous variables or median (25-75th interquartile range) for non-normality variables. Frequency and percentage were used for categorical variables. To analyze the sensitivity and specificity of different BP indexes in relationship to TOD (LVH, eGFR< 60 ml/min per 1.73 m^2^, ACR ≥ 30 mg/g, cIMT≥0.9 mm or carotid plaque), we generated and compared receiver operating characteristic (ROC) curves, including area under the curve (AUC) and their 95% CIs. Considering each TOD may be affected by other important factors, and clinic and ambulatory SBP may have different prognostic value, we established 12 multivariate adjusted logistic regression models in all. All these models in sequence could be divided to four parts according to the TOD categories. Model 1–3, 4–6, 7–9 and 10–12 corresponded with LVH, eGFR< 60 ml/min per 1.73 m^2^, ACR ≥ 30 mg/g, cIMT≥0.9 mm or carotid plaque, respectively. Model 1 included adjustment for age, sex, BMI, smoking, alcohol consumption status, hemoglobin. Albumin, eGFR, number of BP medications and type of glomerular disease. Model 4 included adjustment for age, sex, BMI, smoking, alcohol consumption status. Hemoglobin. albumin, ACR, iPTH, uric acid, calcium* phosphate product, number of BP medications and type of glomerular disease. Model 7 included adjustment for age, sex, BMI, smoking, alcohol consumption status, hemoglobin, albumin, uric acid, number of BP medications and type of glomerular disease. Model 10 included adjustment for age, sex, BMI, smoking, alcohol consumption status, eGFR, LDL-C, statin use, number of BP medications and type of glomerular disease. Model 2,5,8,11 included adjustment for the variables in Model 1,4,7,10 respectively and additional adjustment for clinic SBP when examining 24 h/daytime/nighttime SBP as the independent variable. Model 3,6,9,12 included adjustment for the variables in Model 1,4,7,10 respectively and additional adjustment for nighttime SBP when examining clinic SBP as the independent variable. Odds ratios of clinic SBP, 24-h SBP, daytime SBP and nighttime SBP, were shown per 1 SD increase separately, so as to make them comparable. Probability values were 2-tailed and *P* < 0.05 was considered statistically significant for all comparisons.

## Results

### Demographic and clinical characteristics of the study population

Mean age of the study population was 38.8 years, and 53.7% was male. A total of 752 patients (63.8%) had renal biopsy reports. The number of patients with IgA nephropathy, mesangial proliferative glomerulonephritis (MsPGN), minimal change disease (MCD), membranous nephropathy (MN); focal segmental glomerulosclerosis (FSGS) and membranoproliferative glomerulonephritis (MPGN) was 354, 17, 38, 162, 36 and 9, respectively; 18.5% of patients were current smokers, and101 patients (8.6%) consumed alcohol. The prevalence of LVH, eGFR< 60 ml/min/1.73m^2^, ACR ≥ 30 mg/g, cIMT≥0.9 mm or plaque was 9.8, 38.9, 87.5, 21.3%, respectively (Table 1).

**Table 1:** Demographic characteristics and clinical parameters of study population

Parameters	Value
No. of Patients	1178
Age (years)	38.8 ± 14.0
Male [n(%)]	633 (53.7)
BMI (kg.m^−2^)	22.9 ± 3.5
Smoker [n(%)]	218 (18.5)
Drinker [n(%)]	101 (8.6)
**Primary Glomerular Diseases**	
IgA [n(%)]	354 (30.1)
MsPGN [n(%)]	17 (1.4)
MCD [n(%)]	38 (3.2)
MN [n(%)]	162 (13.8)
FSGS [n(%)]	36 (3.1)
MPGN [n(%)]	9 (0.8)
Others [n(%)]	562 (47.7)
**Medication**	
ACEI or ARB [n(%)]	576 (48.9)
β-blocker [n(%)]	179 (15.2)
CCB [n(%)]	362 (30.6)
α-blocker [n(%)]	71 (6.0)
Statin [n(%)]	200 (17.0)
**Antihypertensive medication use**	
0[n(%)]	347 (29.5)
1[n(%)]	570 (48.4)
2[n(%)]	172 (14.6)
3[n(%)]	78 (6.6)
4[n(%)]	11 (0.9)
**Laboratories**	
Hemoglobin [g/L]	124.8 ± 27.6
Albumin [g/L]	34.5 ± 9.0
Total cholesterol [mmol/L]	5.3 (4.3,6.9)
LDL cholesterol [mmol/L]	3.2 (2.4,4.4)
HDL cholesterol [mmol/L]	1.2 (1.0,1.5)
Triglycerides [mmol/L]	1.5 (1.0,2.3)
Calcium [mg/dL]	8.8 ± 0.8
Phosphate [mg/dL]	3.7 ± 0.4
Calcium* Phosphate [mg^2^/dL^2^]	35.3 ± 9.9
iPTH [pmol/L]	4.9 (3.4,8.5)
Uric acid [μmol/L]	434.0 (346.3522.9)
Creatinine [μmol/L]	97.0 (68.3200.0)
Stage 1[n(%)]	489 (42.4)
Stage 2[n(%)]	231 (19.6)
Stage 3[n(%)]	165 (14.0)
Stage 4[n(%)]	96 (8.1)
Stage 5[n(%)]	197 (16.7)
eGFR-EPI (ml/min/1.73m^2^)	78.0 (30.0,108.0)
eGFR< 60 ml/min/1.73m^2^ [n(%)]	458 (38.9)
ACR [mg/g]	302.4 (85.6851.2)
ACR ≥ 30 mg/g [n(%)]	1031 (87.5)
cIMT-left [mm]	0.7 ± 0.2
cIMT-right [mm]	0.7 ± 0.2
cIMT≥0.9 mm or plaque [n(%)]	251 (21.3)
Left ventricular mass index [g/m^2^]	92.1 ± 24.4
Left ventricular hypertrophy [n(%)]	116 (9.8)

Numbers are mean ± SD, median (25-75th interquartile range) or number (percentage). *MsPGN* Mesangial. proliferative glomerulonephritis, *MCD* Minimal change disease, *MN* Membranous nephropathy, *FSGS* Focal segmental glomerulosclerosis, *MPGN* Membranoproliferative glomerulonephritis, *ACEI* Angiotensin-converting enzyme inhibitor, *ARB* Angiotensin receptor blocker, *CCB* Calcium channel blocker, *LDL* Low-density lipoprotein, *HDL* High-density lipoprotein, *iPTH* Intact parathyroid hormone, *ACR* Albumin-to-creatinine ratio, *cIMT* Carotid. intima-media thickness

### Characteristics of ABPM in the study population

The prevalence of nighttime hypertension in these patients was 62.2%, while 272 patients (23.1%) had isolated nighttime hypertension. A total of 609 (51.7%) patients had non dipper pattern and 197 (16.7%) patients had reversed dipper pattern, while only 341 (28.9%) patients had a dipper pattern. 129(10.9%) of patients had white-coat hypertension and 175 (14.9%) had masked hypertension. (Table 2) Mean BP in each category was shown in supplementary Table 1.

**Table 2:** Clinic and ambulatory blood pressure characteristics in study population

Parameters	Value
Clinic SBP (mmHg)	135.9 ± 23.1
Clinic DBP (mmHg)	85.8 ± 14.4
24 h SBP (mmHg)	126.3 ± 16.7
24 h DBP (mmHg)	78.9 ± 11.3
Daytime SBP (mmHg)	127.8 ± 16.7
Daytime DBP (mmHg)	80.3 ± 11.4
Nighttime SBP (mmHg)	119.6 ± 18.4
Nighttime DBP (mmHg)	73.7 ± 12.7
Nighttime hypertension [n(%)]	733 (62.2)
Isolated nighttime hypertension [n(%)]	272 (23.1)
**Circadian patterns**	
Reverse dipper [n(%)]	197 (16.7)
Non dipper [n(%)]	609 (51.7)
Dipper [n(%)]	341 (28.9)
Extreme dipper [n(%)]	31 (2.6)
**Clinic-ambulatory BP status**	
Normotension [n(%)]	446 (37.9)
White-coat HBP [n(%)]	129 (10.9)
Masked HBP [n(%)]	175 (14.9)
Sustained HBP [n(%)]	428 (36.3)

### Receiver-operating curve analysis of target organ damages

In receiver-operating curve analysis, all SBPs were significantly associated with LVH. Areas under the curve (AUC) were 0.779, 0.770, 0.760, 0.721 for nighttime SBP, 24 h SBP, daytime SBP and clinic SBP respectively. What’s more, nighttime and 24 h SBP ROC curves had greater AUC compared with clinic SBP in detecting the association with LVH (P < 0.05).

When detecting the association with eGFR< 60 ml/min/1.73m^2^, AUC were 0.756, 0.762, 0.756, 0.725 for nighttime SBP, 24 h SBP, daytime SBP and clinic SBP respectively, and statistical analysis showed daytime, nighttime and 24 h SBP had great AUC compared with clinic SBP in detecting the association with eGFR< 60 ml/min/1.73m^2^ (P < 0.05).

When considering ACR ≥ 30 mg/g, AUC were 0.671, 0.654, 0.647, 0.629 for nighttime SBP, 24 h SBP, daytime SBP and clinic SBP respectively, and only nighttime SBP had great AUC compared with clinic SBP in detecting the association with ACR ≥ 30 mg/g by statistical analysis(P < 0.05).

**Fig. 2:**
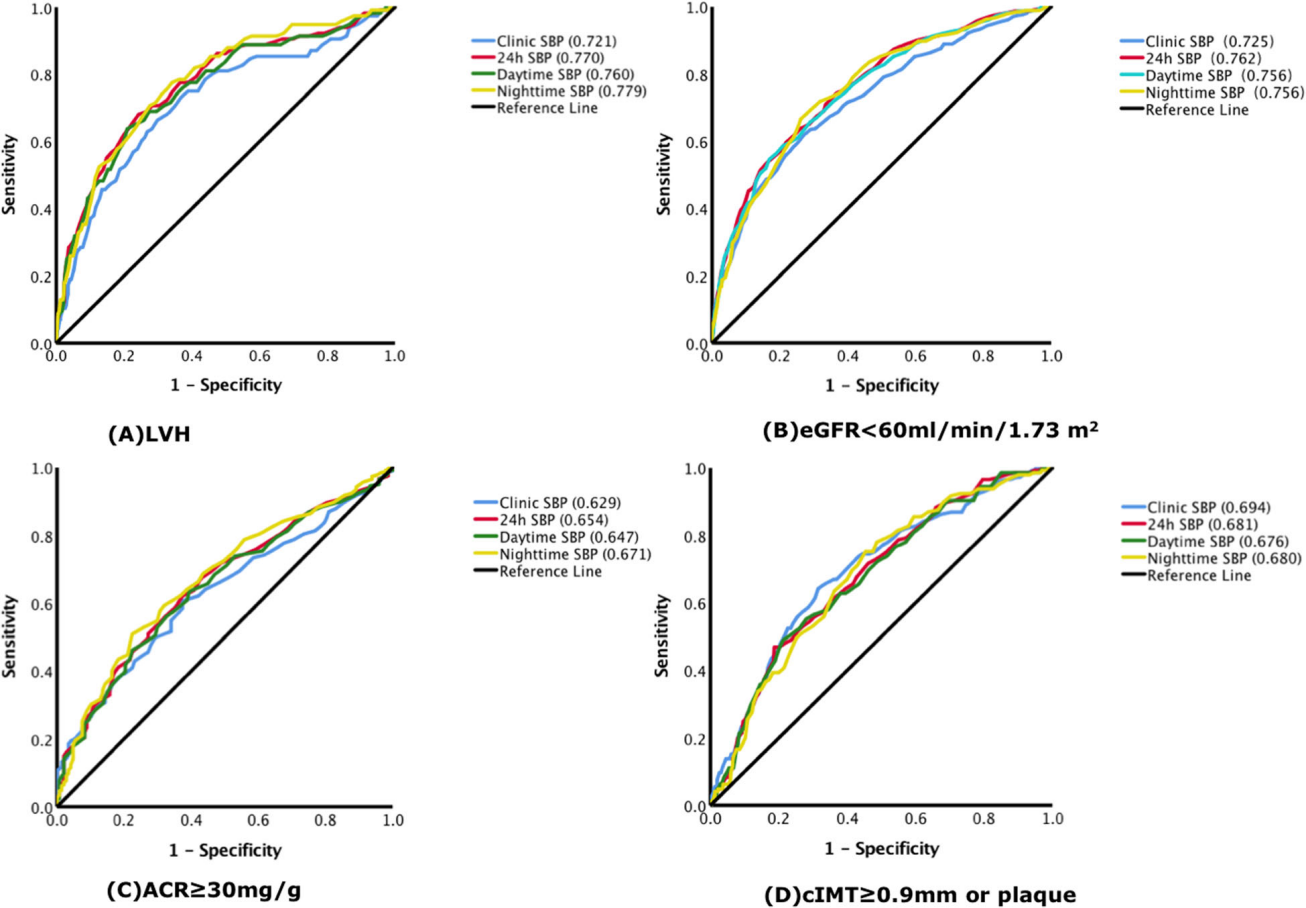
shows receiver operating characteristic(ROC) curves of different BP indexes for TOD in four conditions: **a** left ventricular hypertrophy (LVH): LVMI ≥125 g/m2 (man) or ≥ 120 g/ m2 (woman), **b** eGFR< 60 ml/min per 1.73 m2, **c** ACR ≥ 30 mg/g, (D) cIMT≥0.9 mm or carotid plaque. Value in the bracket is the area under the curve of each line

Finally, when detecting the association with cIMT≥0.9 mm or plaque, AUC were 0.680, 0.681, 0.676, 0.694 for nighttime SBP, 24 h SBP, daytime SBP and clinic SBP respectively, and statistical analysis did not show any difference between ambulatory SBP and clinic SBP in detecting the association with cIMT≥0.9 mm or plaque. (Fig. 2 and Table 3)**.**

**Table 3:** Diagnostic performance of different BP indexes for TOD

	TOD assessments			
	LVH	eGFR<60 ml//min/1.73 m^2^	ACR ≥ 30 mg/g	cIMT≥0.9 mm or plaque
**AUC (95%CI)**				
Clinic SBP	0.721 (0.667, 0.774)	0.725 (0.695, 0.755)	0.629 (0.586, 0.671)	0.694 (0.645, 0.743)
24 h SBP	0.770 (0.722, 0.819)	0.762 (0.734, 0.790)	0.654 (0.610, 0.698)	0.681 (0.632, 0.729)
Daytime SBP	0.760 (0.711, 0.810)	0.756 (0.728, 0.784)	0.647 (0.603, 0.691)	0.676 (0.627, 0.725)
Nighttime SBP	0.779 (0.733, 0.824)	0.756 (0.728, 0.784)	0.671 (0.627, 0.715)	0.680 (0.632, 0.728)
***P*** **value, Z value**				
24 h vs. Clinic SBP	**0.048, 1.977**	**0.007, 2.686**	0.204, 1.272	0.570, 0.568
Daytime vs. Clinic SBP	0.115, 1.578	**0.026, 2.221**	0.368, 0.900	0.441, 0.771
Nighttime vs. Clinic SBP	**0.028, 2.197**	**0.037, 2.083**	**0.045, 2.008**	0.587, 0.543

### Factors associated with target-organ damage by multivariate logistic regression analyses

Multivariate logistic regression analyses were carried out to clarify factors associated with target-organ damage. Higher clinic and ambulatory BPs were significantly associated with higher prevalence of LVH, eGFR< 60 ml/min/1.73m^2^, and ACR ≥ 30 mg/g (***P*** < 0.05). 24 h SBP, daytime SBP and nighttime SBP were still significantly associated with LVH, eGFR< 60 ml/min/1.73m^2^ and ACR ≥ 30 mg/g (***P*** < 0.05) after adjustment by clinic SBP. However, the association of clinic SBP with LVH, eGFR< 60 ml/min/1.73m^2^ and ACR ≥ 30 mg/g (***P*** < 0.05) was attenuated after further adjustment for nighttime SBP (***P*** = 0.290, ***P*** = 0.160, ***P*** = 0.323, respectively). With respect to cIMT≥0.9 mm or plaque, ambulatory SBP or clinic SBP was not significant in multivariate adjusted models with clinic and 24 h/daytime/nighttime SBP included. (Table 4)**.**

**Table 4:** Univariate and multivariate logistic regression analysis of different BP indexes for TOD

	Odds ratio (95% CI), ***P*** value			
	Clinic SBP (per 1 SD)	24 h SBP (per 1 SD)	Daytime SBP (per 1 SD)	Nighttime SBP (per 1 SD)
**LVH**				
Unadjusted	2.130 (1.773, 2.557),< 0.001	2.787 (2.272, 3.420),< 0.001	2.672 (2.184, 3.270),< 0.001	2.720 (2.233, 3.314),< 0.001
Model 1(M1)	1.321 (1.049, 1.665), 0.018	1.598 (1.230, 2.077),< 0.001	1.529 (1.181, 1.980), 0.001	1.624 (1.267, 2.083),< 0.001
Model 2 (M1 + Clinic SBP)	–	1.510 (1.128, 2.021), 0.006	1.432 (1.072, 1.912), 0.015	1.545 (1.186, 2.014), 0.001
Model 3 (M1 + Nighttime SBP)	1.145 (0.891, 1.472), 0.290	–	–	–
**eGFR < 60 ml//min per 1.73 m**^**2**^				
Unadjusted	2.537 (2.189, 2.942),< 0.001	3.003 (2.573, 3.504),< 0.001	2.899 (2.490, 3.376),< 0.001	2.863 (2.455, 3.337),< 0.001
Model 4(M4)	1.265 (0.991, 1.615), 0.059	1.457 (1.145, 1.854), 0.002	1.415 (1.115, 1.796), 0.004	1.492 (1.181, 1.885), 0.001
Model 5 (M4 + Clinic SBP)	–	1.416 (1.073, 1.867), 0.014	1.365 (1.036, 1.798), 0.027	1.446 (1.123, 1.863), 0.004
Model 6 (M4 + Nighttime SBP)	1.089 (0.834, 1.423), 0.529	–	–	–
**ACR ≥ 30 mg/g**				
Unadjusted	1.684 (1.373, 2.065),< 0.001	1.810 (1.474, 2.221),< 0.001	1.753 (1.433, 2.145),< 0.001	1.981 (1.597, 2.457),< 0.001
Model 7(M7)	1.409 (1.080, 1.837), 0.011	1.546 (1.194, 2.002), 0.001	1.501 (1.164, 1.935), 0.002	1.730 (1.326, 2.256),< 0.001
Model 8(M7 + Clinic SBP)	–	1.436 (1.065, 1.935), 0.018	1.384 (1.030, 1.860), 0.031	1.638 (1.231, 2.180), 0.001
Model 9(M7 + Nighttime SBP)	1.156 (0.867, 1.541), 0.323	–	–	–
**cIMT ≥ 0.9 mm or plaque**				
Unadjusted	1.623 (1.413, 1.864),< 0.001	1.517 (1.320, 1.742),< 0.001	1.487 (1.295, 1.708),< 0.001	1.566 (1.365, 1.796),< 0.001
Model 10(M10)	1.088 (0.898, 1.318), 0.389	1.044 (0.856, 1.273), 0.669	1.045 (0.858, 1.273), 0.663	1.070 (0.885, 1.294), 0.482
Model 11 (M10 + Clinic SBP)	–	1.003 (0.800, 1.256), 0.981	1.003 (0.800, 1.256), 0.981	1.044 (0.851, 1.281), 0.681
Model12(M10 + NighttimeSBP)	1.071 (0.870, 1.317), 0.519	–	–	–

M1, M4, M7, M10 were short for Model 1, Model 4, Model 7 and Model 10, respectively. Model 1 included adjustment for age, sex, BMI, smoking, alcohol consumption status, hemoglobin. Albumin, eGFR, number of BP medications and type of glomerular disease. Model 4 included adjustment for age, sex, BMI, smoking, alcohol consumption status. Hemoglobin. albumin, ACR, iPTH, uric acid, calcium* phosphate product, number of BP medications and type of glomerular disease. Model 7 included adjustment for age, sex, BMI, smoking, alcohol consumption status, hemoglobin, albumin, uric acid, number of BP medications and type of glomerular disease. Model 10 included adjustment for age, sex, BMI, smoking, alcohol consumption status, eGFR, LDL-C, statin use, number of BP medications and type of glomerular disease. Model 2,5,8,11 included adjustment for the variables in Model 1,4,7,10 respectively and additional adjustment for clinic SBP when examining 24 h/daytime/nighttime SBP as the independent variable. Model 3,6,9,12 included adjustment for the variables in Model 1,4,7,10 respectively and additional adjustment for nighttime SBP when examining clinic SBP as the independent variable. Odds ratios in the table above present 1 SD increase in SBP

## Discussion

In this cross section study, we explore and compare associations of different BP indexes with TOD in CKD patients with primary glomerular disease. We found that ambulatory SBP, especially nighttime SBP, was better associated with TOD than clinic SBP. What’s more, higher 24 h, daytime and nighttime SBP were significantly associated with TOD in these patients even adjusted clinic SBP in multivariate logistic regression analyses. All these data suggested that ABPM is superior to clinic blood pressure in estimating TOD in patients with primary glomerular disease, and we should pay special attention to the use of ABPM in these patients in clinical practice.

Over the past years, ABPM developed into the recommended technique for BP measurement, risk stratification and classification of hypertension [24, 25]. Compared with clinic BP, ABPM increased the ability to identify circadian variations in BP and identify daytime and nighttime BP. Prior studies have consistently demonstrated significant and superior association of ambulatory SBP with TOD in hypertensive patients [26, 27] as well as in CKD patients [13, 14]^,^. However, all these data were from CKD patients with different causes. CKD patients mixed with different etiologies like primary glomerular disease and diabetic kidney disease, were all included in these studies. Many factors such as glucose, inflammatory, salt intake would affect blood pressure status, so studies enrolled more diabetic patients would draw different conclusion compared with studies enrolled fewer diabetic patients. In previous studies, percentage of patients with diabetic kidney disease or diabetes mellitus at enrollment is up to 65% [12–16]. As the high glucose influences the microenvironment of target organ, including heart, kidney and arteries, patients with diabetic kidney disease showed a more severe TOD, and progressed to ESRD more quickly than other renal disease, once in the period of massive albuminuria. It reminds us of different meanings about ABPM in CKD patients with different etiologies. So we cannot directly extend these conclusions from patients with diabetic and non-diabetic kidney disease to patients with primary glomerular disease.

Primary glomerular disease is still predominant in hospitalized rural patients in China [28]. Data of ABPM in patients with primary glomerular disease was very limited and mostly compared with secondary or diabetic kidney disease in a small sample size [16, 29–32]. Previous study shows that patients with primary glomerular disease have a better control of BP and lower prevalence of abnormal circadian rhythm, when compared with diabetic kidney disease patients [16]. This reminds us probably there’s a difference on the priority of ABPM between CKD patients with and without secondary glomerular disease, especially diabetic kidney disease. Moreover, associations of ABPM with TOD were poorly declared in past studies. Thus, after recruiting a large sample of these population, we evaluate various types of BP measurements, especially ABPM, and assess the strength of their associations with TOD.

The current study strengthened the notion that ABPM, especially nighttime BP carries valuable prognostic information in patients with primary kidney disease. These data confirmed the importance and superiority of ABPM in these patients. Future studies are required to ascertain whether individuals could benefit from BP-lowering interventions targeting the ambulatory monitory results and ultimately reduce cardiovascular events.

Some limitations of our study deserve mention. Firstly, the size of the study population was not very large. Secondly, all enrolled CKD patients underwent only one ABPM and we could not rule out subsequent changes in ABPM. Thirdly, some patients with non-severe proteinuria or renal damage might have been excluded, leading to bias. Finally, we cannot infer a cause–effect relationship based on our cross-sectional data.

## Conclusion

In conclusion, we have provided the first evidence that in CKD patients with primary glomerular disease, 24 h, daytime, and nighttime SBP were better than clinic SBP and significantly associated with TOD, after adjustment for demographics and clinical characteristics. Thus, ABPM should be considered optimal and preferred measurement for estimating cardiovascular risk in these patients.

## Supplementary Information

**Additional file 1: Supplementary Table 1**. Mean blood pressure in each category of study population.

## Data Availability

The datasets used and analyzed during the current study are available from the corresponding author on reasonable request.

## Abbreviations

ABPM: Ambulatory blood pressure monitoring
ACR: Albumin-to-creatinine ratio
ACEI: Angiotensin-converting enzyme inhibitor
ARB: Angiotensin receptor blocker
AUC: Area under the curve
BP: Blood pressure
cIMT: Carotid intima-media thickness
CCB: Calcium channel blocker
CKD: Chronic kidney disease
CKD-EPI: CKD Epidemiology Collaboration
DBP: Diastolic blood pressure
ESRD: End stage renal disease
eGFR: Estimated glomerular filtration rate
FSGS: Focal segmental glomerulosclerosis
HDL: High-density lipoprotein
HBP: High blood pressure
iPTH: Intact parathyroid hormone
LVMI: Left ventricular mass index
LVH: Left ventricular hypertrophy
LDL: Low-density lipoprotein
MsPGN: Mesangial proliferative glomerulonephritis
MCD: Minimal change disease
MN: Membranous nephropathy
MPGN: Membranoproliferative glomerulonephritis
ROC: Receiver operating characteristic
SBP: Systolic blood pressure
TOD: Target organ damage
