# Supplementary material for: Ambulatory blood pressure is better associated with target organ damage than clinic blood pressure in patients with primary glomerular disease
Source: BMC Nephrol. 2020 Dec 11;21:541. doi: 10.1186/s12882-020-02200-1 (PMC7731761; doi:10.1186/s12882-020-02200-1)
Supplement: Supplementary file 1 — Additional file 1: Supplementary Table 1. Mean blood pressure in each category of study population. [file 12882_2020_2200_MOESM1_ESM.docx]

**Supplementary Table 1. Mean blood pressure in each category of study population**

| **Parameters** | **Clinic SBP** | **Clinic DBP** | **24-hour SBP** | **24-hour DBP** |  |
| --- | --- | --- | --- | --- | --- |
| Normotension | 117.6±11.5 | 74.9±7.7 | 112.6±8.3 | 69.4±5.4 |  |
| White-coat HBP | 146.0±16.3 | 91.9±9.7 | 116.7±7.4 | 72.1±5.0 |  |
| Masked HBP | 124.4±10.0 | 80.1±6.6 | 131.9±11.0 | 83.8±7.4 |  |
| Sustained HBP | 156.7±18.7 | 97.6±13.1 | 141.3±12.9 | 89.0±8.5 |  |

Numbers are mean±SD.(mmHg)
